# Correction: Large-scale and fast synthesis of nano-hydroxyapatite powder by a microwave-hydrothermal method

**DOI:** 10.1039/c9ra90034a

**Published:** 2019-05-14

**Authors:** Zhengwei Cai, Xinyu Wang, Zongrui Zhang, Yingchao Han, Jing Luo, Mingzheng Huang, BoWen Zhang, Yuanjing Hou

**Affiliations:** State Key Laboratory of Advanced Technology for Materials Synthesis and Processing, Wuhan University of Technology Wuhan 430070 China wangxinyu@whut.edu.cn +86-27-87880734 +86-27-87651853; Biomedical Materials and Engineering Research Center of Hubei Province Wuhan 430070 China; College of Biochemical Engineering, Anhui Polytechnic University Wuhu 241000 China

## Abstract

Correction for ‘Large-scale and fast synthesis of nano-hydroxyapatite powder by a microwave-hydrothermal method’ by Zhengwei Cai *et al.*, *RSC Adv.*, 2019, **9**, 13623–13630.

The Acknowledgement section contained an incorrect funder number. The correct version of the acknowledgements is shown below:

This work was supported by the National Key R&D Program of China (No. 2017YFC1103800); the Science and Technology Partnership Program, Ministry of Science and Technology of China (KY201602002); the major special project of Technological Innovation of Hubei Province (No. 2017ACA168).

The Royal Society of Chemistry apologises for these errors and any consequent inconvenience to authors and readers.

## Supplementary Material

